# Factors Predicting Trial Engagement, Treatment Satisfaction, and Health-Related Quality of Life During a Web-Based Treatment and Social Networking Trial for Binge Drinking and Depression in Young Adults: Secondary Analysis of a Randomized Controlled Trial

**DOI:** 10.2196/23986

**Published:** 2021-06-07

**Authors:** Samineh Sanatkar, Milena Heinsch, Peter Andrew Baldwin, Mark Rubin, Jenny Geddes, Sally Hunt, Amanda L Baker, Kathryn Woodcock, Terry J Lewin, Kathleen Brady, Mark Deady, Louise Thornton, Maree Teesson, Frances Kay-Lambkin

**Affiliations:** 1 Centre for Brain and Mental Health Research School of Medicine and Public Health The University of Newcastle Callaghan Australia; 2 The Matilda Centre for Research in Mental Health and Substance Use The University of Sydney Camperdown Australia; 3 Black Dog Institute UNSW Sydney Randwick Australia; 4 School of Psychology The University of Newcastle Callaghan Australia; 5 Psychiatry and Behavioral Sciences Medical University of South Carolina Charleston, SC United States

**Keywords:** digital mental health, personality, negative affect, study engagement, life quality

## Abstract

**Background:**

Mental health and alcohol use problems are among the most common causes of disease burden in young Australians, frequently co-occur (comorbidity), and lead to significant lifetime burden. However, comorbidities remain significantly underdetected and undertreated in health settings. Digital mental health tools designed to identify at-risk individuals, encourage help-seeking, or deliver treatment for comorbidity have the potential to address this service gap. However, despite a strong body of evidence that digital mental health programs provide an effective treatment option for a range of mental health and alcohol use problems in young adults, research shows that uptake rates can be low. Thus, it is important to understand the factors that influence treatment satisfaction and quality-of-life outcomes for young adults who access e–mental health interventions for comorbidity.

**Objective:**

In this study, we seek to understand the factors that influence treatment satisfaction and quality-of-life outcomes for young adults who access e–mental health interventions for comorbid alcohol and mood disorders. The aim is to determine the importance of personality (ie, Big Five personality traits and intervention attitudes), affective factors (ie, depression, anxiety, and stress levels), and baseline alcohol consumption in predicting intervention trial engagement at sign-up, satisfaction with the online tool, and quality of life at the end of the iTreAD (Internet Treatment for Alcohol and Depression) trial.

**Methods:**

Australian adults (N=411) aged between 18 and 30 years who screened positive for depression and alcohol use problems signed up for the iTreAD project between August 2014 and October 2015. During registration, participants provided information about their personality, current affective state, alcohol use, treatment expectations, and basic demographic information. Subsequent follow-up surveys were used to gauge the ongoing trial engagement. The last follow-up questionnaire, completed at 64 weeks, assessed participants’ satisfaction with web-based treatment and quality-of-life outcomes.

**Results:**

Multiple linear regression analyses were used to assess the relative influence of predictor variables on trial engagement, treatment satisfaction, and quality-of-life outcomes. The analyses revealed that the overall predictive effects of personality and affective factors were 20% or lower. Neuroticism constituted a unique predictor of engagement with the iTreAD study in that neuroticism facilitated the return of web-based self-assessments during the study. The return of incentivized follow-up assessments predicted treatment satisfaction, and state-based depression predicted variance in quality-of-life reports at study completion.

**Conclusions:**

Our findings suggest that traditional predictors of engagement observed in face-to-face research may not be easily transferable to digital health interventions, particularly those aimed at comorbid mental health concerns and alcohol misuse among young adults. More research is needed to identify what determines engagement in this population to optimally design and execute digital intervention studies with multiple treatment aims.

**Trial Registration:**

Australian New Zealand Clinical Trials Registry (ACTRN): 12614000310662; http://www.anzctr.org.au/Trial/Registration/TrialReview.aspx?id=365137&isReview=true.

**International Registered Report Identifier (IRRID):**

RR2-10.1186/s12889-015-2365-2

## Introduction

### Background

Mental health and alcohol use disorders are the leading causes of disability among young adults globally [1,2]. This trend is reflected in Australia, with alcohol misuse and poor mental health reported as the primary contributors to disease burden among Australian youth, including young adults [3]. The burden of disease increases when substance use and mental health disorders co-occur. For example, comorbidity of alcohol use disorder and major depressive disorder is associated with elevated risks of alcohol dependence, higher instances of attempted suicide, and lower levels of functioning and life satisfaction [4,5]. Consequently, treatment prognoses for this population tend to be relatively poor [6,7].

Complicating the path to recovery, young Australians in their teens and 20s see their general practitioners or mental health professionals less frequently than their older counterparts [8]. Recent research found that across 1306 adults presenting for assessment of their general practitioners, comorbid alcohol use and major depressive disorders were correctly detected in only 21% of cases [9]. This situation highlights the need to improve the identification of comorbid disorders because high nondetection rates constitute a major barrier to access appropriate and timely treatment.

Digital technologies offer a promising opportunity to identify comorbid disorders and enhance access to high-quality mental health care for young adults [10,11]. Digital services can function as stand-alone programs or in conjunction with face-to-face or web-based clinical support. These types of services are acceptable to young people and align with recent findings that young people use digital apps for numerous purposes (including supporting their health and well-being) at a greater rate than any other age group [12]. Previous research indicates that 44% of Australians aged between 16 and 25 years use the internet to access health information [13]. A recent review found that face-to-face help-seeking among young people aged between 12 and 25 years may improve after using computerized mental health programs and services [14]. However, sustained engagement with digital interventions has proved difficult, and limited participation and high attrition rates are common [11]. This is concerning because the amount of exposure to an intervention is often an important prerequisite for intervention outcomes [15].

Studies that have evaluated engagement in digital health interventions suggest that it may be an important contributor to participant satisfaction and quality of life at the conclusion of a research study or treatment program. For example, in a post hoc analysis of a randomized controlled trial evaluating the effectiveness of an online support group on primary care patients’ mental health, Geramita et al [16] found that those who engaged more frequently with a study-specific online support group reported significant improvements in health-related quality of life 6 months after the trial phase had ended compared with participants who had engaged the least. Consequently, evaluating engagement with mental health treatment protocols has been identified as critical for improving intervention impact and success [17]. Although engagement with digital interventions is not identical to engagement in clinical trials of these interventions, dropout rates in clinical trials can reflect disengagement rates with digital interventions in naturalistic settings when sample bias and trial *push factors* are kept to a minimum [18-20].

Person-based characteristics can predict engagement with health treatment protocols and intervention success in younger adult samples. In particular, personality patterns, such as high levels of conscientiousness and low levels of neuroticism, predicted adherence to study protocols in trials for smoking cessation therapy, asthma control, and health-related quality of life [21-23], whereas negative emotional states and unfavorable attitudes toward treatments can negatively influence engagement and treatment satisfaction displayed by young adults in clinical trials across a variety of health conditions [24-29]. Personality patterns of low conscientiousness, high neuroticism, and extraversion have consistently been associated with problematic levels of alcohol consumption among adolescents and young adults [30], indicating that personality may influence both the formation of and recovery from health-related complications in young adulthood. This highlights the usefulness of considering young adults’ personality, affect, and treatment beliefs as predictors of engagement with study protocols. Clarifying the unique role played by each of these factors may help to remedy the poor uptake of web-based mental health interventions [11,31].

### Objectives

This study aims to examine the relative importance of personality factors (ie, conscientiousness and neuroticism), negative emotional states (ie, stress, anxiety, and depression), recent alcohol use, and treatment expectations in predicting engagement with a web-based mental health study designed to reduce depressive symptoms and incidences of heavy episodic drinking (*binge drinking*) in young adulthood. In addition, we examined the predictive strength of personality, negative emotions, alcohol consumption, treatment expectations, and study engagement on participants’ self-reported treatment satisfaction and quality of life at the end of the web-based mental health study. Specifically, it was hypothesized that a pattern of low neuroticism, high conscientiousness, lower emotional distress, lower alcohol consumption, and greater expectations of treatment success at sign-up would predict higher levels of subsequent study engagement, treatment satisfaction, and quality-of-life ratings at the last assessment point. It was further predicted that higher study engagement levels during the trial would predict greater treatment satisfaction and quality of life at the last assessment point. To our knowledge, this is the first study to assess these predictors simultaneously within a randomized controlled trial design that evaluates the effectiveness of a web-based mental health program to reduce the severity of comorbid substance use disorder and major depression among young Australian adults. In doing so, our study seeks to contribute to the literature on web-based mental health program acceptance among young adults in line with the recommendations of Clarke et al [11].

## Methods

### Overview

The iTreAD (Internet Treatment for Alcohol and Depression) trial study protocol was prospectively registered with the Australian and New Zealand Clinical Trials Registry (ACTRN12614000310662) and approved by the UNSW Sydney Human Research Ethics Committee (HC13299). The protocol has been previously published [32].

### Participants and Procedures

Recruitment to iTreAD occurred between August 2014 and October 2015. Following an initial screener, a total of 421 eligible candidates from Australia (aged between 18 and 30 years) who reported current depressive symptoms and alcohol misuse and had internet access completed the informed consent process and baseline survey and were randomly allocated to one of three conditions: (1) web-based self-assessments, (2) web-based self-assessments with an additional digital mental health program, or (3) web-based self-assessments, digital mental health programs, and an additional clinician-guided digital forum (refer Kay-Lambkin et al [32] for further details on the study design). In total, 10 participants subsequently withdrew their consent to participate in the iTreAD study and were removed from the analysis. The final sample consisted of 411 participants, of which 135 were allocated to the web-based self-assessment control group, 131 to the web-based self-assessment and digital mental health program group, and 145 to the joint web-based self-assessment, digital mental health program, and clinician-guided forum group. Most participants were female (252/411, 61.3%) and had a mean age of 23 (SD 3.67) years.

The overall study duration was 64 weeks. During this time, all participants were asked to complete 12 monthly web-based self-assessments and four follow-up surveys at 26, 39, 52, and 64 weeks after their initial baseline assessment. Participants were reimbursed up to Aus $20 (US $15.44) for their baseline assessment and for each follow-up assessment they completed, regardless of participation rates in any of the three study conditions. No reimbursement was provided for the 12-monthly web-based self-assessments. Only participants allocated to either of the two treatment groups gained access to their respective web-based intervention components.

### Measures

#### Personality

Conscientiousness was measured using the 9-item *Conscientiousness* subscale of the Big Five Inventory (BFI) [33], which asks participants to rate their agreement with statements such as, “I see myself as someone who does a thorough job.” Neuroticism was measured using the 2-item *Neuroticism* subscale of the BFI short form [34], which asks participants to rate their agreement with statements such as, “I see myself as someone who gets nervous easily.” Both the long and short forms of the BFI have been found to be reliable and valid measures of the Big Five trait dimensions [33-36].

#### Negative Affective States

Recent experiences of negative affective states were measured using the Depression, Anxiety, and Stress Scale-21 items (DASS-21) [37]. The DASS-21 has been demonstrated to have good psychometric properties in clinical, nonclinical, and adolescent samples and has been used in previous research assessing affective states of web-based intervention users [38-40]. An example item for depression is, “I couldn’t seem to experience any positive feeling at all.”

#### Alcohol Consumption

Heavy drinking and probable alcohol dependence were assessed using the 3-item Consumption Short Form of the Alcohol Use Disorders Identification Test-Concise (AUDIT-C) [41]. The AUDIT-C is a valid and reliable measure to screen for possible alcohol use disorders similar to the AUDIT long form [41,42]. An example item is, “How often did you have six or more drinks on one occasion?” with responses ranging from 0 (*never*) to 4 (*daily*). Overall AUDIT-C scores range from 0 to 12, with a score of 3 or above warranting further assessment of whether an alcohol use disorder may be present.

#### Treatment Expectations and Satisfaction

Treatment expectations at the beginning of the study were measured by asking participants, “By the end of treatment, how much improvement in binge drinking and depression do you think could occur as a result of internet delivered treatment?” Participants responded to this question in increments of 10 points ranging from the lowest (0-10) to highest expected improvement (91-100). Treatment satisfaction at the end of the study was measured using the general 8-item Client Satisfaction Questionnaire [43], which has demonstrated concurrent validity among Australians seeking treatment for alcohol and substance abuse [44]. An example item for client satisfaction is, “In an overall general sense, how satisfied are you with the service you received?”

#### Study Engagement

We considered returned web-based self-assessments (0-12) and follow-up questionnaires (0-4) as indicators of participants’ engagement with the iTreAD study. Web-based self-assessments were part of the iTreAD treatment protocol because reflecting on one’s recent mood and alcohol consumption was thought to have a mild therapeutic effect [45], and the completion of follow-up questionnaires constituted an indicator of study adherence. Thus, the self-assessments and follow-up questionnaires captured elements of treatment and study protocol engagement in the iTreAD trial. No other engagement indices were shared across the experimental and control groups.

#### Quality of Life

Participants’ health-related quality of life was assessed using the 20-item standard version of the Assessment of Quality of Life questionnaire (AQoL-6D) [46]. The instrument considers various physical and psychological indicators of quality of life and general functioning. The validity of its components has been confirmed in Australian adult samples [47]. An example item is, “How often do you feel in control of your life?” Higher scores on the AQoL-6D were indicative of *lower* perceived quality of life.

### Analytic Plan

With regard to the primary outcomes of the iTreAD trial, multilevel regression models revealed significant decreases in depression severity and binge drinking episodes throughout the study; however, these improvements did not vary by treatment condition (Kay-Lambkin et al, unpublished data, April 2021). This pattern of results suggested that there were no systematic differences between groups in the outcome measures, either at baseline or as a result of any intervention. Therefore, we collapsed the three trial conditions across all outcome measures to retain a sufficiently powered sample size for the analysis regarding study engagement. After collapsing across groups, 390 participants completed all relevant measures at registration. A total of 190 participants completed the treatment satisfaction questionnaire, and 191 participants completed the quality-of-life assessment at the last assessment point at 64 weeks. This reduction in available data was because of the study attrition and optional completion of the questionnaire components. Using G*Power 3.1 (Heinrich Heine University Düsseldorf) [48], power calculations indicated that a sample size of 205 was required to detect small-to-medium effects typical in personality research with α of .05, a power of 0.80, and 9 predictor variables. These power calculations indicated that the treatment satisfaction and quality-of-life analyses were slightly underpowered and would be more suitable for detecting medium-sized effects.

Predictor variables were identified based on the literature, indicating a likely impact on engagement. These variables were the personality traits of conscientiousness and neuroticism, the negative affective states of depression, anxiety, stress, alcohol use, and treatment expectations at the outset of the study. The BFI subscales of neuroticism and conscientiousness were used to describe personality trait dimensions. The DASS-21 subscales of depression, anxiety, and stress were used as predictor variables of negative affect. AUDIT-C scores were used to indicate alcohol consumption levels, and the single-item measure “By the end of treatment, how much improvement in binge drinking and depression do you think could occur as a result of internet delivered treatment?” assessed at baseline was used as a proxy for treatment expectations. The total number of returned study questionnaires (ie, web-based self-assessments and follow-up questionnaires) were computed to describe indices of study engagement. The sum scores of the 8-item Client Satisfaction Questionnaire and AQoL-6D were used to measure treatment satisfaction and quality of life, respectively, at the end of the study.

Multiple linear regression analyses were performed using SPSS Statistics for Windows, version 25 (IBM Corporation) to assess the extent to which baseline levels of personality, negative affect, alcohol consumption, and treatment expectations might predict subsequent study engagement and to assess the ability of personality, negative affect, alcohol consumption, treatment expectations, and study engagement to predict treatment satisfaction and quality of life at the end of the study. The resulting prediction models reveal the unique and combined contributions of the predictor variables that help explain the proportion of the total variance of each outcome variable. As we considered three outcomes, we tested a total of three model predictions.

## Results

### Sample Characteristics

Table 1 presents the descriptive and between-group statistics of the iTreAD study participants. There were no significant differences in key variables between groups, including treatment satisfaction at the end of the study (mean 23.90, SD 5.17; *F*_2,196_=2.14; *P*=.12), except for engagement. On average, participants in the control condition returned around two additional questionnaires (mean 6.51, SD 4.19) from the web-based self-assessment component compared with participants in experimental conditions 1 (mean 3.75, SD 3.87) and 2 (mean 4.43, SD 4.19; *F*_2,408_=16.59; *P*<.001). It is possible that these differences were because of limited engagement options in the control condition (ie, questionnaire assessment only), whereas the experimental conditions offered web-based activities in addition to the questionnaire completion options.

**Table 1:** Scale response anchors: means, SDs, and between-group statistics on key variables.

Variable	Scale anchors	Control group (n=135)			Experimental group 1 (n=131)			Experimental group 2 (n=145)			*F* test (df)		*P* value
	Range	Mean (SD)	n^a^ (%)	Mean (SD)		n^a^ (%)	Mean (SD)		n^a^ (%)				
Age (years)^b^	18-30	23.24 (3.59)	129 (95.6)	23.2 (3.67)		128 (97.7)	23.68 (3.74)		134 (92.4)	0.71 (2, 388)		.494	
Conscientiousness	1-5	3.35 (0.66)	128 (94.8)	3.25 (0.65)		128 (97.7)	3.26 (0.66)		138 (95.2)	0.82 (2, 391)		.44	
Neuroticism	1-5	3.84 (1.06)	127 (95.1)	3.87 (1.02)		128 (97.7)	3.84 (1.08)		138 (95.2)	0.03 (2, 390)		.97	
Depression	0-42	25.22 (9.53)	133 (98.5)	26.43 (8.5)		130 (99.2)	25.74 (9.40)		144 (99.3)	0.58 (2, 404)		.56	
Anxiety	0-42	16.72 (8.78)	133 (98.5)	17.88 (8.74)		130 (99.2)	17.97 (9.87)		144 (99.3)	0.78 (2, 404)		.46	
Stress	0-42	23.56 (8.30)	133 (98.5)	24.97 (8.51)		130 (99.2)	24.71 (8.49)		144 (99.3)	1.05 (2, 404)		.35	
Alcohol consumption	0-12	7.66 (1.99)	135 (100)	7.55 (2.16)		131 (100)	7.57 (2.05)		143 (98.6)	0.11 (2, 406)		.90	
Treatment expectation	0-100	46.23 (22.04)	130 (96.3)	47.44 (21.77)		129 (98.5)	45.07 (22.48)		138 (95.2)	0.38 (2, 394)		.68	
Self-assessments	0-12	6.51 (4.19)	135 (100)	3.75 (3.87)		131 (100)	4.43 (4.19)		145 (100)	16.59 (2, 408)		<.001	
Follow-ups	0-4	2.35 (1.61)	135 (100)	1.92 (1.73)		131 (100)	2.14 (1.66)		145 (100)	2.21 (2, 406)		.11	
Treatment satisfaction^c^	8-32	22.9 (5.72)	72 (53.3)	24.56 (4.84)		57 (43.5)	24.39 (4.73)		70 (48.3)	2.14 (2, 196)		.12	
Quality of life^d^	20-99	39.51 (9.63)	73 (54.1)	40.28 (11.24)		57 (43.5)	41.27 (11.67)		70 (48.3)	0.48 (2, 197)		.62	

^a^Responses to items were optional. Actual numbers ranged from 57 to 145 per group.

^b^Age, personality, emotional distress, and treatment expectation statistics were computed at registration.

^c^Treatment satisfaction and quality of life were assessed at trial completion after 64 weeks postbaseline.

^d^Lower scores on the quality-of-life measure indicate higher life quality.

Table 2 lists Pearson correlations between variables. Conscientiousness showed consistent weak-to-moderate negative correlations with neuroticism, depression, anxiety and stress levels, and alcohol consumption (*r* ranged between −0.20 and −0.26) and was associated with higher levels of study engagement (the correlation with self-assessment returns was *r*=0.12 and with follow-up returns was *r*=0.11) and higher reported quality of life at the end of iTreAD at 64 weeks (*r*=−0.25). Conversely, higher initial reported stress and anxiety levels as well as alcohol consumption were negatively associated with web-based self-assessment returns (*r*=−0.15 and *r*=−0.12, respectively), and higher initial levels of alcohol consumption were negatively associated with returns of follow-up assessments (*r=*−0.14). Neuroticism and negative affect at baseline showed moderate correlations with decreased quality of life at the 64-week follow-up (*r* ranged between 0.23 and 0.41). In addition, lower perceived quality of life was associated with lower levels of treatment satisfaction at the 64-week follow-up (*r*=−0.20). Treatment satisfaction was positively correlated with the number of returned follow-up assessments (*r=*0.18) in this study.

**Table 2:** Pearson correlations between key variables^a^.

Variable			1		2		3		4		5		6		7		8		9		10
**1. Conscientiousness**																					
	*r*	—^b^		—		—		—		—		—		—		—		—		—	
	*P* value	—		—		—		—		—		—		—		—		—		—	
**2. Neuroticism**																					
	*r*	−0.24		—		—		—		—		—		—		—		—		—	
	*P* value	<.001		—		—		—		—		—		—		—		—		—	
**3. Depression**																					
	*r*	−0.24		0.20		—		—		—		—		—		—		—		—	
	*P* value	<.001		<.001		—		—		—		—		—		—		—		—	
**4. Anxiety**																					
	*r*	−0.26		0.44		0.45		—		—		—		—		—		—		—	
	*P* value	<.001		<.001		<.001		—		—		—		—		—		—		—	
**5. Stress**																					
	*r*	−0.20		0.46		0.53		0.69		—		—		—		—		—		—	
	*P* value	<.001		<.001		<.001		<.001		—		—		—		—		—		—	
**6. Alcohol consumption**																					
	*r*	−0.12		−0.06		0.21		0.11		0.12		—		—		—		—		—	
	*P* value	.02		.21		<.001		.02		.02		—		—		—		—		—	
**7. Treatment expectation**																					
	*r*	0.08		−0.00		0.07		0.08		0.13		−0.07		—		—		—		—	
	*P* value	.10		.96		.18		.13		.01		.18		—		—		—		—	
**8.** **Self-assessments**																					
	*r*	0.12		0.05		−0.07		−0.15		−0.12		−0.08		0.05		—		—		—	
	*P* value	.02		.35		.18		.003		.02		.10		.32		—		—		—	
**9. Follow-ups**																					
	*r*	0.11		0.03		−0.03		−0.10		−0.06		−0.14		0.08		0.70		—		—	
	*P* value	.03		.56		.49		.05		.22		.006		.09		<.001		—		—	
**10. Treatment satisfaction**																					
	*r*	0.04		0.05		−0.04		0.06		0.04		−0.11		0.14		−0.01		0.18		—	
	*P* value	.54		.53		.56		.43		.54		.14		.05		.90		.01		—	
**11. Quality of life^c^**																					
	*r*	−0.25		0.23		0.41		0.35		0.40		0.08		−0.01		−0.05		−0.13		−0.20	
	*P* value	<.001		.001		<.001		<.001		<.001		.25		.93		.44		.08		.005	

^a^Personality, emotional distress, and treatment expectation statistics were computed at registration; treatment satisfaction and quality of life were assessed at trial completion 64 weeks postbaseline. Responses to items were optional. Actual n ranged between 192 and 415.

^b^Not applicable.

^c^Lower scores on the quality-of-life measure indicated higher life quality.

### Regression Models

For model 1, personality, negative affect, alcohol consumption, and treatment expectation variables were included as predictors of iTreAD study engagement, as indicated by the return of web-based self-assessments (Table 3) and follow-up questionnaires (Table 4). The models predicting self-assessment (*F*_7,382_=3.09; *P*=.003) and follow-up returns (*F*_7,382_=2.61; *P*=.01) were significant. However, the predictors only accounted for about 5% of the variance in study engagement (*R^2^*=0.05), indicating that personality, negative affect, alcohol consumption, and expectations of treatment success together played a minor role in determining continuous study participation. Neuroticism remained a statistically significant predictor of study engagement, although its unique predictive power was low. Neuroticism alone accounted for approximately 2% of the variance in web-based self-assessments. In other words, an increase of about one point (0.71) in the neuroticism dimension was predictive of returning an additional web-based self-assessment.

**Table 3:** Standard regression results for personality, negative affect, and treatment expectations predicting engagement with web-based self-assessments (n=390)^a^.

Model	*b* (SE)	β	Pearson *r*	*sr* ^b^	Structure coefficient
Constant	1.81 (1.93)	N/A^c^	N/A	N/A	N/A
Conscientiousness	0.67 (0.34)	.10	0.12	0.10	0.500
Neuroticism^d^	0.71 (0.24)	.18	0.05	0.15	0.203
Depression	0.02 (0.03)	.04	−0.07	0.03	−0.289
Anxiety	−0.07 (0.03)	−.14	−0.14	−0.10	−0.595
Stress	−0.05 (0.04)	−.10	−0.11	−0.07	−0.474
Alcohol consumption	−0.06 (0.11)	−.03	−0.11	−0.03	−0.319
Treatment expectation	0.01 (0.01)	.07	0.05	0.06	0.228

^a^*R^2^*=0.05; adjusted *R^2^*=0.04.

^b^*sr*: the semipartial correlation.

^c^N/A: not applicable.

^d^*P*<.01.

**Table 4:** Standard regression results for personality, negative affect, and treatment expectations predicting engagement with follow-up questionnaires (n=390)^a^.

Model	*b* (SE)	β	Pearson *r*	*sr* ^b^	Structure coefficient
Constant	1.81 (0.75)	N/A^c^	N/A	N/A	N/A
Conscientiousness	0.23 (0.13)	.09	0.11	0.09	0.519
Neuroticism	0.18 (0.09)	.12	0.04	0.10	0.164
Depression	0.01 (0.01)	.04	−0.05	0.03	−0.243
Anxiety	−0.02 (0.01)	−.09	−0.09	−0.07	−0.435
Stress	−0.01 (0.02)	−.06	−0.07	−0.04	−0.313
Alcohol consumption	−0.08 (0.04)	−.10	−0.13	−0.10	−0.617
Treatment expectation	0.01 (0.00)	.08	0.08	0.08	0.393

^a^*R^2^*=0.05, adjusted *R^2^*=0.03.

^b^*sr*: the semipartial correlation.

^c^N/A: not applicable.

Model 2 (presented in Table 4) examined the predictive strength of personality, negative affect, treatment expectations, and study engagement on participants’ satisfaction with the iTreAD program. Contrary to expectations, the overall model only approached significance (*F*_9,180_=1.90; *P*=.06), indicating that the variance of satisfaction with the treatment program could not be explained by the prediction model. However, the study engagement variable of follow-up assessments showed a significant association with treatment satisfaction in the overall model. The return of follow-up assessments explained 2.5% of the variance in treatment satisfaction, whereby the completion of about 2 (1.6) additional questionnaires was predictive of a 1-point increase in treatment satisfaction.

Personality, negative affect, treatment expectations, and study engagement were used to predict the self-reported quality of life at study cessation in model 3 (Tables 5 and 6). The prediction model was significant (*F*_9,181_=6.34; *P*<.001). The combined predictors accounted for approximately 20% of the variance in quality of life (*R^2^*=0.24; adjusted *R^2^*=0.20). Only depression scores at baseline had a significant influence on subsequent quality-of-life ratings and, given the other variables in the model, only accounted for about 3% of the variance in quality of life. In other words, a 0.3-point increase on the DASS-21 depression measure uniquely accounted for a 1-point increase on the AQoL-6D (ie, a 1-unit decrement in life quality).

**Table 5:** Standard regression results for personality, negative affect, treatment expectations, and study engagement predicting treatment satisfaction (n=190)^a^.

Model	*b* (SE)	β	Pearson *r*	*sr* ^b^	Structure coefficient
Constant	16.78 (3.88)	N/A^c^	N/A	N/A	N/A
Conscientiousness	0.32 (0.63)	.04	0.06	0.04	0.194
Neuroticism	0.23 (0.43)	.05	0.05	0.04	0.180
Depression	−0.06 (0.05)	−.11	−0.07	−0.09	−0.252
Anxiety	0.03 (0.06)	.06	0.05	0.04	0.167
Stress	0.03 (0.07)	.04	0.03	0.03	0.099
Alcohol consumption	−0.10 (0.19)	−.04	−0.09	−0.04	0.310
Treatment expectation	0.03 (0.02)	.13	0.13	0.13	0.446
Self-assessments	−0.14 (0.11)	−.11	−0.00	−0.10	0.003
Follow-ups^d^	1.63 (0.54)	.25	0.20	0.22	0.677

^a^*R^2^*=0.09; adjusted *R^2^*=0.04.

^b^*sr*: the semipartial correlation.

^c^N/A: not applicable.

^d^*P*<.01.

**Table 6:** Standard regression results for personality, negative affect, treatment expectations, and study engagement predicting quality of life (n=191)^a^.

Model	*b* (SE)	β	Pearson *r*	*sr* ^b^	Structure coefficient
Constant	38.40 (7.23)	N/A^c^	N/A	N/A	N/A
Conscientiousness	−1.94 (1.17)	−.12	−0.25	−0.11	−0.502
Neuroticism	0.65 (0.81)	.06	0.25	0.05	0.506
Depression^d^	0.30 (0.09)	.27	0.39	0.22	0.802
Anxiety	0.08 (0.11)	.07	0.34	0.05	0.688
Stress	0.17 (0.13)	.14	0.39	0.08	0.788
Alcohol consumption	−0.19 (0.36)	−.04	0.10	−0.03	0.202
Treatment expectation	−0.03 (0.03)	−.06	−0.01	−0.06	−0.014
Self-assessments	0.19 (0.20)	.07	−0.04	0.06	0.084
Follow-ups	−1.74 (1.01)	−.13	−0.16	−0.11	−0.331

^a^*R*^2^=0.24; adjusted *R*^2^=0.20; lower scores on the quality-of-life measure indicated higher quality of life.

^b^*sr*: the semipartial correlation.

^c^N/A: not applicable.

^d^*P*<.01.

## Discussion

### Principal Findings

We hypothesized that a combination of personality traits (conscientiousness and neuroticism), recent feelings of negative affect (depression, anxiety, and stress), recent alcohol use, and expectations of treatment effectiveness could predict subsequent study engagement within a young adult–focused web-based intervention trial (iTreAD). We further hypothesized that personality, negative affect, alcohol use, treatment expectations, and study engagement would predict treatment satisfaction and self-reported quality of life at the end of iTreAD. We expected that conscientiousness, treatment expectations, and study engagement would exert a positive influence on study engagement and outcomes, whereas neuroticism, negative affect, and greater alcohol consumption at baseline would pose obstacles to study engagement and derive any intended benefit. This study sought to extend the existing literature by combining these predictive factors into a single model to test the overall magnitude of influence and discern the unique contributions of predictor variables.

As there were no previous studies known to us that had assessed predictors of engagement, treatment satisfaction, and quality-of-life outcomes simultaneously, we had no a priori expectations regarding the shared and unique contributions of predictor variables in explaining outcomes. Our results indicated that the combined effects of personality, negative affect, alcohol use, and attitudes in predicting study engagement were relatively low. Although conscientiousness, anxiety, stress, and alcohol consumption showed significant zero-order correlations with our measures of study engagement, only 5% of the variance in study engagement exhibited by young Australian adults could be attributed to personality, negative affect, alcohol use, and treatment expectations at the time of study registration, when considering all predictor variables. This is a clinically important finding, indicating that this range of presenting characteristics supporting engagement in mental health services in previous research was not as salient in a population of young adults with comorbid depression and alcohol use problems. It is well recognized that comorbid substance use and mental disorders are among the strongest factors associated with nonengagement with mental health treatment [49], and young people represent a particular subgroup of our community who are among the hardest to engage in treatment [6]. Although treatment engagement is a complex and multidimensional issue, a review of people with mental illness highlighted the potential for digital tools to remove many of the traditional barriers to access mental health treatment, to encourage ongoing psychoeducation, to outreach to people in environments in which they feel comfortable and safe, and to promote autonomy and empowerment [6]. These issues may be particularly important for young people considering treatment for sensitive mental health problems and for people with comorbidities who may experience service fragmentation and disconnection when seeking care. It may be that the digital environment offered our study population an opportunity that overcame some of these typical predictors of engagement. Future research should seek to understand digital tools in this context, particularly given that our study still reported high levels of attrition over time.

Among the predictors of study engagement, and contrary to expectations, only elevated neuroticism levels at baseline seemed to be associated with increased numbers of monthly web-based self-assessments returned. Previous studies demonstrated that neuroticism could indeed be beneficial for performance because of individuals’ increased tolerance for negative affect [50], which may have played a supportive role in participants’ willingness to complete self-assessments in this study. These findings resemble what previous researchers have coined *healthy neuroticism*, where a combination of neurotic self-awareness and conscientious decision making indicates engaging in favorable health behaviors such as increased doctor visits and fewer risky behaviors such as alcohol consumption and smoking [51-54]. Importantly, Weston and Jackson [52] found that *healthy neurotics* were more successful than other personality types in implementing effective behavior change *after* disease onset, suggesting that awareness of a chronic condition facilitated healthful actions particularly well in this group. Hence, it is possible that among treatment-seeking young people, moderate levels of neuroticism are beneficial to increase the chances of ongoing engagement with a digital mental health intervention study.

Concerning treatment satisfaction, the prediction model did not yield statistical significance, indicating that our predictors did not explain substantive variance in treatment satisfaction. Reductions in the adjusted R-squared value compared with the initial R-squared value indicated that the variables entered were in fact detrimental to explaining variance in treatment satisfaction. Although the overall model did not reach the statistical level of significance, it is worth noting that, in line with zero-order correlations, completion of incentivized follow-up assessments was uniquely predictive of satisfaction ratings at the conclusion of the study. Participants were invited to complete follow-up assessments 26, 39, 52, and 64 weeks after study registration and were reimbursed each time they returned a follow-up assessment. It is possible that the ongoing reflection on symptom scores combined with positive reinforcement through the incorporation of a tangible monetary reward system facilitated satisfaction with the study overall. This may hint toward the utility of supervision and blended care models, where some clinician guidance takes place alongside digital mental health therapy. Furthermore, personality, emotional distress, the severity of alcohol consumption, and even treatment allocation did not seem to affect satisfaction with the trial negatively. Satisfaction ratings with the study were good across all the conditions. These results warrant further exploration of factors influencing young adults’ satisfaction with web-based treatment components in particular, given the increasing focus on providing web-based health tools to young, digitally native populations [14].

Most notably, personality, affect, treatment expectation, and engagement variables together predicted about one-fifth of the variance in quality-of-life responses at the end of the iTreAD study. Although there were numerous moderate-sized zero-order associations with quality of life, when all predictors were included within one model (and with this, the sizable correlations between the predictor variables were accounted for), only depression remained a unique contributor to the variance in quality of life. These results underscore that depressive symptoms are a robust contributor to poor functioning in young people facing problems with alcohol and deserve attention in the design and delivery of digital mental health interventions.

### Strengths and Limitations

Although the strength of this analysis lies in the simultaneous inspection of predictive factors, several limitations warrant consideration. First, our study’s engagement measures were somewhat limited in scope. Although our study engagement variables captured both treatment and trial engagement, only one common aspect of the treatment protocol (monthly web-based self-assessments) could be considered for the analysis. Ideally, web-based treatment engagement metrics would comprise several treatment components, such as the number of log-ins and module completion rates within the digital mental health intervention [55]. Power requirements prevent such an approach in our analyses; however, future research should attempt to uncover person-based predictors of web-based treatment engagement using more content-valid engagement measures. Second, although the decision as to which variables to include in this analysis was informed by the existing literature, the results revealed that the overall contribution of personality, affect, alcohol use, and expectations in predicting markers of treatment success were small, indicating that other, more important factors determined engagement in this digital treatment trial. Third, because of the optional completion of study components and study attrition, analyses including variables assessed at the conclusion of the study (ie, treatment satisfaction and quality of life) were slightly underpowered and thus needed to be interpreted with caution.

### Conclusions

Taken together, our findings suggest that web-based mental health trials should continue to consider and aim to treat initial levels of depression to optimally improve quality-of-life experiences at the conclusion of the intervention period. In addition, neuroticism may constitute a positive predictor of subsequent engagement with the treatment protocol, and study adherence incentivized through monetary rewards may indicate improved satisfaction with the digital service overall.

Digital mental health interventions have the potential to become an integral part of health promotion strategies aimed at young people [11]. However, to meet this objective, web-based treatments need to capture their target audience’s demands and deliver health information and mental health support in a comprehensive, effective, and engaging way. Our findings suggest that traditional predictors of engagement observed in face-to-face and even some web-based research may not be easily transferable to evaluate digital health interventions, particularly those aimed at comorbid mental health concerns and alcohol misuse among young adults.

Future research should continue to assess which factors and their combinations reliably and substantially predict young adults’ engagement with digital mental health tools. As a next step, similar to face-to-face psychotherapy recommendations [56], supervised and tailored approaches to digital health and mental health promotion may yield the most engaging prospect as interventions can be personalized and delivered in a manner that suits the person undertaking web-based treatment [15].

## Abbreviations

AUDIT-C: Alcohol Use Disorders Identification Test-Concise
AQoL-6D: Assessment of Quality of Life questionnaire
BFI: Big Five Inventory
DASS-21: Depression, Anxiety, and Stress Scale-21 items
iTreAD: Internet Treatment for Alcohol and Depression
